# CircPTK2 (hsa_circ_0005273) as a novel therapeutic target for metastatic colorectal cancer

**DOI:** 10.1186/s12943-020-1139-3

**Published:** 2020-01-23

**Authors:** Hongbao Yang, Xiaobo Li, Qingtao Meng, Hao Sun, Shenshen Wu, Weiwei Hu, Guilai Liu, Xianjing Li, Yong Yang, Rui Chen

**Affiliations:** 1 State Key Laboratory of Natural Medicines, Institute of Pharmaceutical Science, China Pharmaceutical University, Nanjing, 211198 China; 2 Key Laboratory of Environmental Medicine Engineering, Ministry of Education, School of Public Health, Southeast University, Nanjing, 210009 China; 3 School of Public Health, Advanced Innovation Center for Human Brain Protection, Capital Medical University, Beijing, 100069 People’s Republic of China; 4 Beijing Key Laboratory of Environmental Toxicology, Capital Medical University, Beijing, 100069 People’s Republic of China; 5 School of Pharmacy, Xuzhou Medical University, 209 Tongshan Road, Xuzhou, 221004 Jiangsu China

**Keywords:** CRC, circRNA, circPTK2, EMT, Vimentin

## Abstract

**Background:**

As a novel class of noncoding RNAs, circRNAs have been recently identified to regulate tumorigenesis and aggressiveness. However, the function of circRNAs in colorectal cancer (CRC) metastasis remains unclear. We aimed to identify circRNAs that are upregulated in CRC tissues from patients and study their function in CRC metastasis.

**Methods:**

We compared six pairs of CRC tissues and their matched adjacent non-tumor tissues by using circRNA microarray. We first evaluated the expression of circPTK2 (hsa_circ_0005273) in fresh tissues from CRC tumors and corresponding adjacent tissues by qPCR analysis. CircPTK2 expression levels in the tissue microarray with 5 years of survival information were determined by RNA-ISH analysis. Meanwhile, the expression levels of circulating circPTK2 were further analyzed according to the patients’ clinical features. We analyzed cell apoptosis, colony formation, migration, and invasion in CRC cells. To further elucidate the effect of circPTK2 in CRC metastasis, we also conducted a colon cancer hepatic and pulmonary metastasis experiment. We used RNA biotin-labeled pull down and mass spectrometry to identify the target of circPTK2. We established a PDTX model to evaluate the effect of shRNA specifically targeting circPTK2 on tumor metastasis.

**Results:**

We identified a novel circRNA, circPTK2, which is back-spliced of three exons (exons 27, 28 and 29) of PTK2 by using circRNA microarray, bioinformatics and functional studies. CircPTK2 was elevated in CRC tissues and positively associated with tumor growth and metastasis. CRC patients with increased circPTK2 expression were positively correlated with poorer survival rates. Furthermore, our studies showed that circPTK2 could promote EMT of CRC cells in vitro and in vivo by binding to vimentin protein on sites Ser38, Ser55 and Ser82. We further demonstrated the interaction of circPTK2 and vimentin mediated the regulation of CRC by knockdown or overexpression of vimentin. In addition, we revealed that tail vein injection of shRNA specifically targeting circPTK2 blunt tumor metastasis in a patient-derived CRC xenograft model.

**Conclusions:**

Collectively, these results demonstrate that circPTK2 exerts critical roles in CRC growth and metastasis and may serve as a potential therapeutic target for CRC metastasis, and also a promising biomarker for early diagnosis of metastasis.

## Background

Colorectal cancer (CRC) is the third most common and second leading cause of cancer death in the world [1]. Although significant progress has been made in the treatment of CRC in recent decades, the prognosis is still poor, especially in the distant metastasis of advanced-stage tumors [2, 3]. Therefore, finding novel potential biomarkers for early diagnosis and prediction of CRC metastasis and further elucidating the precise molecular mechanisms can provide improved treatment for CRC patients.

Circular RNAs (circRNAs), a class of non-coding RNAs, are characterized by a covalent bond linking the 3’and 5′ ends generated by backsplicing [4]. Most circRNAs result from a non-canonical form of alternative splicing, a process mediated by spliceosomes or by group I and group II ribozymes [5]. Genome-wide analyses of RNA sequencing data have showed evolutionary conservation and abundance of circular RNAs [6, 7]. CircRNAs can be generated from exons, called exonic circular RNAs [6, 8], or from introns, called intronic circular RNAs, or a mix of both called circRNAs [9, 10]. CircRNAs have been found to function as endogenous miRNA sponges since some circRNAs possess miRNA binding sites, allowing them to arrest miRNA activity [11]. Besides, circRNAs also function as RNA-binding protein (RBP) sequestering agents as well as transcription regulators to modulate gene expressions [12–14]. Emerging evidences suggest that circRNAs are closely related to human diseases, especially cancers, and can serve as better biomarkers because of their abundance and stability. Recently, several circRNAs have been found to be associated with CRC tumorigenesis or metastasis, such as circCCDC66, hsa_circ_001569, ciRS-7-A, and circHIPK3 [15–19]. These studies demonstrate that the regulation of circRNA expression is tightly controlled under distinct circumstances, and that the investigations into circRNA are still in their infancy. The functions and underlying mechanisms of circRNAs in the epithelial–mesenchymal transition (EMT) process of human cancers, such as CRC, remain unclear. EMT is the key to tumor metastasis [20, 21]. EMT converts epithelial cells to a mesenchymal-like phenotype and is associated with the loss of cell contacts, production of the type-III intermediate filament protein vimentin, and increases in cell migration and invasion [22].

In this study, we identified a molecular effector mechanism of circPTK2 (hsa_circ_0005273 in circBase: http://www.circbase.org) in CRC using circRNA microarray, bioinformatics, and functional studies. We found that circPTK2 was elevated in CRC tissues and positively associated with tumor growth and metastasis. CRC patients with increased circPTK2 expression were positively correlated with poorer survival rates. Further mechanism studies showed that circPTK2 could promote EMT of CRC cells in vitro and in vivo by targeting vimentin protein on sites Ser38, Ser55 and Ser82. We further demonstrated that the interaction of circPTK2 and vimentin mediated the regulation of CRC by knockdown or overexpression of vimentin. In addition, we showed that tail vein injection of shRNA specifically targets circPTK2 blunt tumor metastasis in a patient-derived CRC xenograft model. In concert, we identified that circPTK2 is a promising biomarker for early diagnosis of CRC metastasis and provides a potential therapeutic target for CRC.

## Methods

### Patients and specimens

All samples used in this study were in compliance with the informed consent and agreement of patients as well as the ethical regulations (No. 2017ZDKYSB165) of Southeast University, China.

Fresh tumor tissue and corresponding adjacent noncancerous tissues were collected from CRC patients, which included a test set (consisting of 61 CRC cases enrolled from the Jiangsu Tumor Hospital) and a validation set (consisting of 131 CRC cases enrolled from the Affiliated Hospital of the Xuzhou Medical College), between 2014 and 2016. Tumor stage was defined according to the criteria of the sixth edition of TNM classification of the International Union Against Cancer (UICC, 2009). Detailed information of CRC patients is presented in Additional file 7: Table S1.

To determine whether circRNA serves as a blood-based early biomarker for CRC detection, peripheral blood samples were collected simultaneously with the tumor tissues mentioned above. Meanwhile, peripheral blood samples were also collected from sex- and age-matched healthy donors enrolled from the Nanjing Hospital of Chinese Medicine from 2014 to 2016. Criteria for healthy control exclusion included those with a history of diabetes, obesity (BMI over 30), hypertension (systolic blood pressure over 120 mm of mercury (mm Hg) and/or diastolic blood pressure over 80 mmHg), clinically diagnosed chronic cardiovascular diseases, chronic kidney diseases, chronic gastrointestinal diseases, and respiratory diseases. Detailed information of healthy control is presented in Additional file 7: Table S1. Because the total RNA extracted from six samples was unqualified, and six blood samples were not collected, these samples were not counted in. Therefore, the sample size finally included in the statistics is 186.

For the construction of tissue microarray (TMA), the CRC and adjacent non-tumor tissues (5 cm from the tumoral margins) were collected from two independent cohorts as previously reported [23]. The test cohort (376 patients) was recruited from the Affiliated Hospital of Xuzhou Medical College between 2007 and 2011. A validation cohort consisting of 702 patients was recruited from the Jiangsu Tumor Hospital between 2007 and 2011. All of the patients were followed-up by a trained clinical specialist through in-person or family contact from the time of diagnosis to death, or last follow-up (the last follow-up was in June 2016). The maximum follow-up time was 112.7 months and the median survival time (MST) was 75.0 months. Detailed information on these patients is listed in Additional file 8: Table S2 [23]. Because 105 samples were lost during antigen retrieval or without relevant cells present in the core, the number of sample size finally included in the statistics is 973.

### Cell culture and in vitro experiments

Human CRC cell lines (HCT15, SW620, SW480 and LOVO) and 293 T were purchased from American Type Culture Collection (ATCC) (Manassas, VA).

Details are provided in Additional file 10.

### CircRNA microarray

Six pairs of CRC tissues (3 colon and 3 rectal carcinomas) and the corresponding adjacent noncancerous tissues, as well as six adenomas (all tubulovillous adenomas) were utilized for circRNA microarrays. The specimens were obtained from patients undergoing surgery in the Jiangsu Tumor Hospital in 2015; detailed information is shown in Additional file 9: Table S3.

Details are provided in Additional file 10.

### Tissue/serum preparation and RNA isolation

Details are provided in Additional file 10.

### RNAscope

circPTK2 expression levels in CRC cells, fresh paraffin-embedded tissues, and TMA were assayed with the BaseScopeTM Reagent Kit v2-RED (Advanced Cell Diagnostics, Newark, CA) according to the manufacturer’s instructions. Briefly, samples were incubated with circPTK2 probes (NM_153831.3, nt2804–2485) at 40 °C for 2 h. For the fresh paraffin-embedded tissues, the samples were rehydrated for 15 min and digested with protease for 30 min before incubation with the probe. After hybridization, a series of single amplification procedures were performed. circPTK2 expression levels were ultimately visualized with Fast Red.

### CircRNA in situ hybridization (RNA-ISH)

Tissue microarray (TMA) slides were de-paraffinized using xylene for three 5-min washes; rehydrated with an ethanol gradient via 5-min washes in 100, 95, and 80% ethanol; and then treated with 20 μg/mL Proteinase K (Roche Diagnostics, Indianapolis, IN) for 10 min at 37 °C. After that, the slides were fixed in 4% formaldehyde (Thermo Scientific, Rockford, IL) for 10 min, rinsed twice using 0.13 M 1-methylimidazole, and re-fixed with 1-ethyl-3-(3-dimethylaminopropyl) carbodiimide (EDC, Thermo Scientific) for 1 h. The 1% H_2_O_2_ was used to block endogenous peroxidases, and the slides were pre-hybridized at 50 °C for 30 min in the hybridization buffer [50% formamide (American Bioanalytical)], 5 × SSC (American Bioanalytical, Natick, MA), 50 μg/mL heparin (Sigma-Aldrich, St. Louis, MO), 0.1% Tween 20 (Sigma-Aldrich, St. Louis, MO), and 500 μg/mL yeast tRNA (Invitrogen, Carlsbad, CA), with a pH = 6. Next, the slides were hybridized with 200 nM double digoxigenin (DIG) LNA modified.

The staining of circPTK2 in the TMA was scored independently by two pathologists blinded to the clinical data using the following criterion: Category A: the intensity of immunostaining was scored from 0 to 3 (0, negative; 1, weak; 2, moderate; and 3, strong); Category B: the percentage of immunoreactive circPTK2 was deemed as 1 (0–25%), 2 (26–50%), 3 (51–75%), and 4 (76–100%). The circPTK2 score was calculated by multiplying Category A and Category B.

### In vivo experiments

All animal experiments were conducted with the approval of the Center for New Drug Evaluation and Research, China Pharmaceutical University (Nanjing, China) in a 12-h light and 12-h dark turnover environment, and in accordance with the National Institutes of Health Guide for the Care and Use of Laboratory Animals. The animals were housed in standard pathogen-free (SPF) conditions at 24 °C ± 2 °C, with 40 to 70% relative humidity. Water and a basal diet were given ad libitum. The nude mice were purchased from the Model Animal Research Center of Nanjing University (China). Of note, 5 × 10^6^ cells less than 15 passage after the initial drug selection were implanted in the flank site of 6-week-old female nude mice (*n* = 3/group). Fourteen days later, images were taken by an in vivo imaging system (IVIS) Spectrum (PerkinElmer, USA). The tumor, liver, and lung tissues were harvested, and the biochemical luciferase activities in the homogenate of tissues were determined by a luminometer (Sirius, Berthold Detection Systems, Germany).

### Quantitative real-time PCR analysis

For mature circRNA expression analysis, 1 μg of total RNA was converted to cDNA using a One Step PrimeScript® circRNA cDNA Synthesis Kit (Takara, Shiga, Japan). After reverse transcription, quantitative real-time PCR analysis was performed using the SYBR Premix Ex Taq kit (Takara, Shiga, Japan) along with the specific circRNA LNA™ PCR primer sets designed by Exiqon (Denmark). GAPDH was used as a normalization control for all of the samples. All of the experiments were performed in triplicates. The primers were 5′-AATGCCTGTGAACCCATAGTG-3′ (forward) and 5′- CTGACAGCATGAGCATCCCT − 3′ (reverse) for circPTK2.

### Lentivirus stable transduction

Lentiviruses were generated by co-transfection of the expression vector of interest with the packaging plasmids psPAX2 and pMD2G.

Details are provided in Additional file 10.

### Western blotting

For western blotting assays, proteins isolated from tissues were incubated with primary antibody detecting vimentin and E-cadherin (1: 1000 dilution; cell signaling), and β-actin (1:1000 dilution; cell signaling) was used as a control.

Details are provided in Additional file 10.

### siRNA, shRNA, and plasmid construction and cell transfection

siRNAs targeting circPTK2 and vimentin were designed and synthesized by RiboBio (Guangzhou, China).

Details are provided in Additional file 10.

### Immunohistochemistry (IHC) staining

Tissues were fixed with 4% paraformaldehyde, dehydrated, embedded in paraffin and sectioned at 4 μm. Sections were deparaffinized, rehydrated and incubated with 3% H_2_O_2_. After antigen repair and being blocked, the slides were incubated with rabbit monoclonal antibody against vimentin (1:100) (Cell Signaling Technology, USA) at 4 °C overnight. Subsequently, the slides were incubated with secondary antibody at room temperature for 30 min and then incubated with streptavidin peroxidase complex. Staining was performed using 3, 3-diaminobenzidine (DAB) substrate kit for peroxidase reaction and counterstained with hematoxylin. Finally, the slides were analyzed with a light microscope.

### Biotin-labeled RNA pull-down and mass spectrometry analysis

Biotin-labeled RNA for liner sequence of circPTK2 was generated by an in vitro transcription reaction with the Biotin RNA Labeling Mix (Roche, Mannheim, Germany) and T7 RNA polymerase (Roche, Mannheim, Germany), and then treated with RNase-free DNase I (Takara, Japan). After incubation with guide oligonucleotide targeting circular junction, the liner probe was then circularized using T4 RNA ligase I, treated with RNase R. After purified with RNeasy Mini Kit (Qiagen, Inc., Valencia, CA, USA), the biotin-labeled RNA probe (3 μg) was then incubated with cell extracts from CRC cells at room temperature (RT) for 2 h, and treated with 35 μl of Streptavidin C1 magnetic beads (Invitrogen) for 1 h. after washed, the retrieved protein was detected by western blot or mass spectrometry analysis (CapitalBio Technology, Beijing, China).

### Patient-derived tumor xenograft (PDTX) models

Briefly, fresh human CRC tissues were washed twice with cold PBS containing penicillin (500 U/mL) and streptomycin (500 μg/mL), and transplanted subcutaneously into the right flanks of NCG mice. When the tumor volume reached 250 mm^3^ (V = 0.5 × length × width^2^), xenografts were resected and passaged into additional NCG mice. Successfully xenografted CRC tumor models were passaged and banked after three passages in vivo. For isolation of the PDTX tumor cells, xenografts were disaggregated mechanically, and washed twice using PBS with penicillin (500 U/mL), streptomycin (500 μg/mL), gentamicin (100 mg/L), and amphotericin B (2.5 mg/L). Next, the tumor mass was digested in culture medium containing type II/IV collagenase (1 mg/mL) and DNase (1 mg/mL) at 37 °C for 1 h with gentle shaking. After filtration through a 80 μm filter (BD Biosciences, San Jose, CA), the cell suspension was centrifuged at 300 g for 5 min at 4 °C. The pellets were resuspended using Dulbecco’s Modified Eagle Medium (DMEM) and seeded in 6-well plates for further experimentation. Subsequently, mice bearing lower circPTK2-expressing xenografts were injected with control and circPTK2-overexpressing lentivirus by tail vein, whereas mice bearing higher circPTK2-expressing xenografts were injected with control shRNA and circPTK2 shRNA lentivirus by tail vein. Twenty-one days later, mice were killed and liver sections were subjected to H&E staining and autofluorescence assay.

### Analysis of cell colony formation, invasion, migration ability, and apoptosis

Details are provided in Additional file 10.

### Statistical analysis

Data are shown as mean ± standard error of the mean (SE). The 2^-ΔΔ Ct^ method was used to analyze the results of real-time PCR in all of the experiments. Differences in the circRNA levels of the tissues were analyzed using the Wilcoxon rank-sum test. The receiver operating characteristic (ROC) curves plotted by R package’s “pROC” were applied to estimate the diagnostic value of CIRC on CRC detection [Robin X, et al. pROC: an open-source package for R and S+ to analyze and compare ROC curves. BMC Bioinformatics 12, 77 (2011)].

The time-dependent ROC curve analysis was used to calculate the best cutoff value of circRNA. The different values of the follow-up time were evaluated through the performances of different scores by plotting (t, AUC[T]). The probability of difference in overall survival (OS) was ascertained by Kaplan-Meier curves, and the significance was detected by a log-rank test. Univariate or multivariate Cox regression analysis was applied to estimate the crude hazard ratios (HRs), adjusted HRs, and corresponding 95% confidence intervals (CIs). Statistical analysis was performed using SAS software 9.4, GraphPad Prism, and significance was set at *P* < 0.05.

## Results

### Expression profiles of circRNAs in CRC

**Fig. 1:**
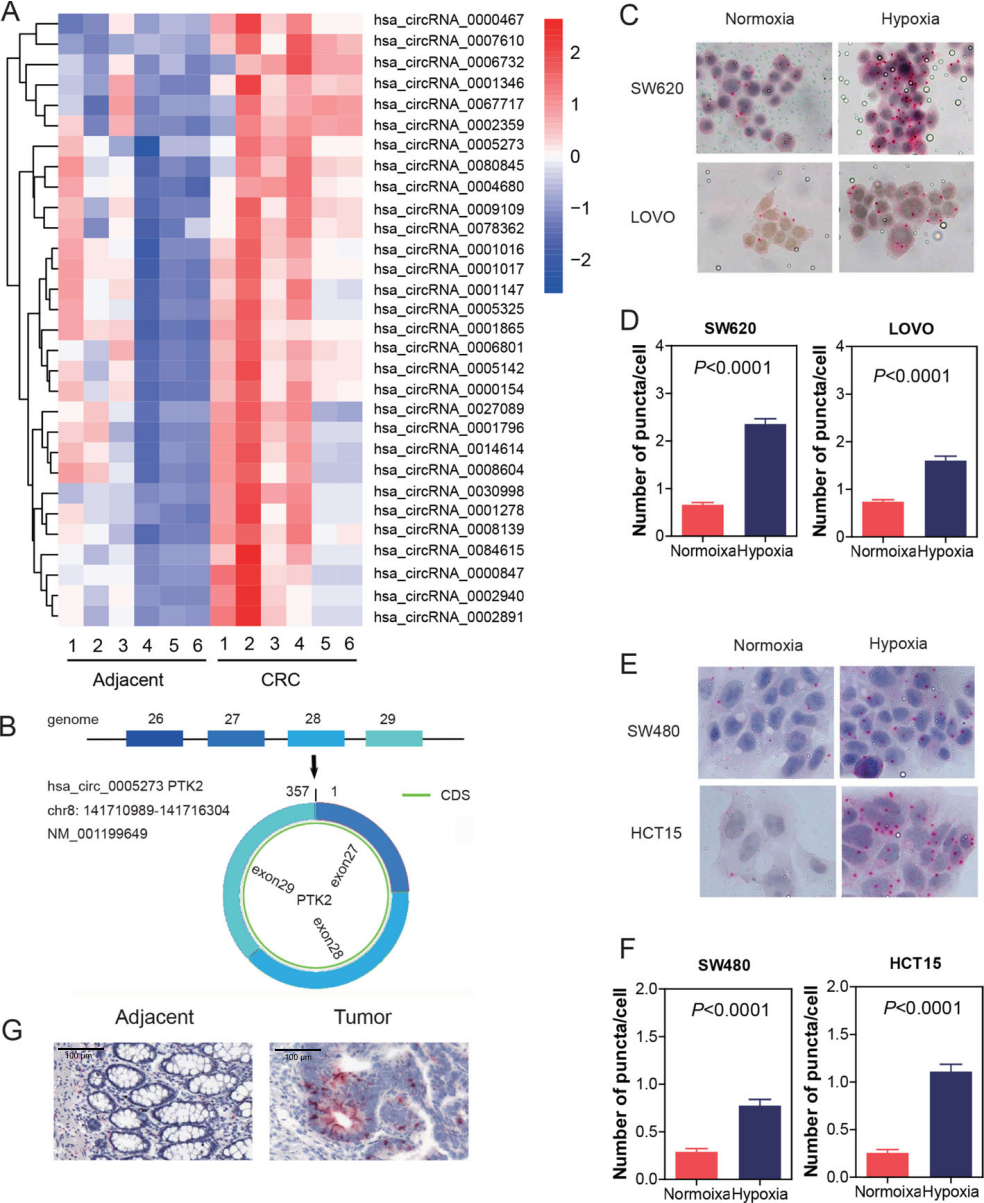
Expression profiles of circRNAs in CRC and characterization of circPTK2. **a** Heatmap of the differentially expressed circRNAs in six pairs of human CRC tissues and adjacent non-tumor tissues. Red: upregulated circRNAs in CRC; green: downregulated circRNAs in CRC. **b** circPTK2 is back-spliced by exons 27, 28, and 29 of PTK2. **c**, **d** Cells were treated with hypoxia. circPTK2 was detected by RNAscope. Location and expression of circPTK2 in SW620 and LOVO cells. **e**, **f** Location and expression of circPTK2 in SW480 and HCT15 cells. **g** Location and expression of circPTK2 in adjacent and tumor of patient tissue. *P* values were calculated by one-way ANOVA

To characterize circRNA expression profiles in CRC tissues, we compared six pairs of CRC tissues and their matched adjacent non-tumor tissues by using circRNA microarray. As a result, 30 circRNAs (*P* < 0.05 and fold change > 2.0) and their host gene mRNAs (*P* < 0.05 and fold change > 1.5) were upregulated between the CRC tumor tissues and paired adjacent normal tissues. The top 30 upregulated circRNAs were shown by hierarchical clustering (Fig. 1a).

In this study, circPTK2 (chr8:141710989-141716304) was estimated as the seventh highly upregulated circRNA in CRC, with a more than six-fold change from microarray analysis. By browsing the human reference genome (GRCh37/hg19), we identified that circPTK2 is back-spliced of three exons (exons 27, 28, and 29) of PTK2 (Fig. 1b). The human gene PTK2, encoding FAK, is localized at chromosome 8q24.3, a region characterized by frequent aberrations in human cancers. Focal adhesion kinase (FAK) is a multifunctional regulator of cell signal transduction in the tumor microenvironment. FAK promotes cell migration, survival, and proliferation through kinase-dependent and -independent mechanisms in various tumors. Elevated levels of FAK are related to the progression of multiple malignant tumors, and its role in regulating cell proliferation, migration, and apoptosis has been extensively studied [24–27].

We detected the localization of circPTK2 in cells or tissues. Using RNAscope ISH, we found that circPTK2 existed in both cytoplasm and nucleus (Fig. 1c and e). Hypoxia is one of the most common stressors encountered within the tumor microenvironment. Here, we used hypoxia to mimic the tumor microenvironment and detected the expression or location alteration under the hypoxic condition. We found that the expression of circPTK2 significantly accumulated after hypoxic induction, whereas the location was not changed, whether in high-level circPTK2 cells (SW620 or LOVO cells) or low-level circPTK2 cells (SW480 or HCT15 cells) (Fig. 1c, d, e, and f). Interestingly, results from tissues showed that circPTK2 existed only in tumor tissues, not in normal tissues (Fig. 1g).

### CircPTK2 is elevated in both CRC tissues and serum and associated with tumor metastasis

**Fig. 2:**
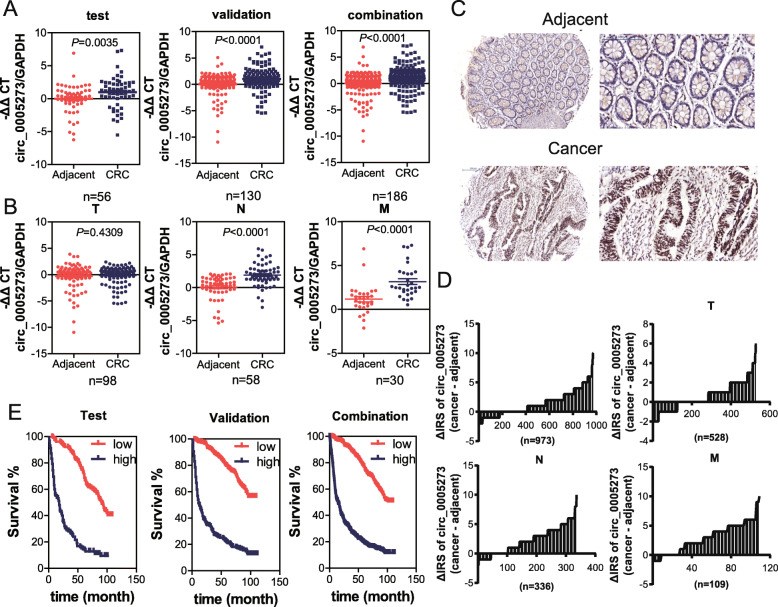
CircPTK2 is overexpressed in tissues of CRC patients and associated with tumor metastasis. **a** Levels of circPTK2 in the indicated fresh tissues from the testing (*n* = 56), validation (*n* = 130), and the combination set (*n* = 186) were detected by qPCR. The circPTK2 expression was normalized to GAPDH. **b** The abundance of circPTK2 in CRC patients with different clinical characteristics were further analyzed. T: primary tumor; N: node metastasis; M: distant metastasis. Data are presented as -ΔΔCt by one-way ANOVA with three technical replicates each. **c** The levels of circPTK2 in TMA were evaluated by RNA-ISH analysis, and the representative staining images are shown. **d** The difference in staining score between CRC lesions and adjacent non-tumor tissues in total (*n* = 973), tumor (T) (*n* = 528), node (N) (*n* = 285) and metastasis (M) (*n* = 110) cohort are shown. **e** Kaplan–Meier curves depicts the overall survival of CRC patients according to the tumoral circPTK2 levels. Left to right: testing cohort, validation cohort and combination cohort. *P* values were calculated by the log-rank test

To determine whether circPTK2 participates in CRC, we first evaluated the expression of circPTK2 in fresh tissues from CRC tumors (CRC) and corresponding adjacent tissues. qPCR analysis showed that the abundance of circPTK2 in CRC tissues was markedly elevated compared to adjacent tissue in the testing, validation, and combination sets (Fig. 2a). Moreover, dramatically higher tumoral circPTK2 levels were found in patients with lymph node or distal metastases (Fig. 2b). No significant difference in circPTK2 levels was detected in patients with primary tumors (Fig. 2b). Furthermore, circPTK2 expression levels in the TMA with 5 years of survival information (containing 1078 pairs of CRC tissues and matched adjacent noncancerous tissues) were determined by RNA-ISH analysis. As shown in Fig. 2c and d, circPTK2 levels were significantly overexpressed in CRC tissues compared with adjacent non-tumor tissues. In addition, higher tumoral circPTK2 levels correlated with shorter OS time (Fig. 2e).

Meanwhile, the levels of serumal circPTK2 expression were further analyzed according to the patients’ clinical features. Consistent with the results in tissues, the abundance of circPTK2 in CRC serum was markedly elevated compared to the serum from healthy controls (NOR) in the testing, validation, and combination sets (Additional file 1: Figure S1A). In addition, patients with node or distal metastases exhibited the elevated abundance of circPTK2 in serum (Additional file 1: Figure S1B). Furthermore, ROC analysis showed that circPTK2 is indicative of CRC in patients with node or distal metastases (Additional file 1: Figure S1C). Taken together, our results indicate that tumoral circPTK2 is associated with poor clinical features, including metastasis, in CRC patients.

### CircPTK2 promoted aggressive phenotypes of CRC cells in vitro

**Fig. 3:**
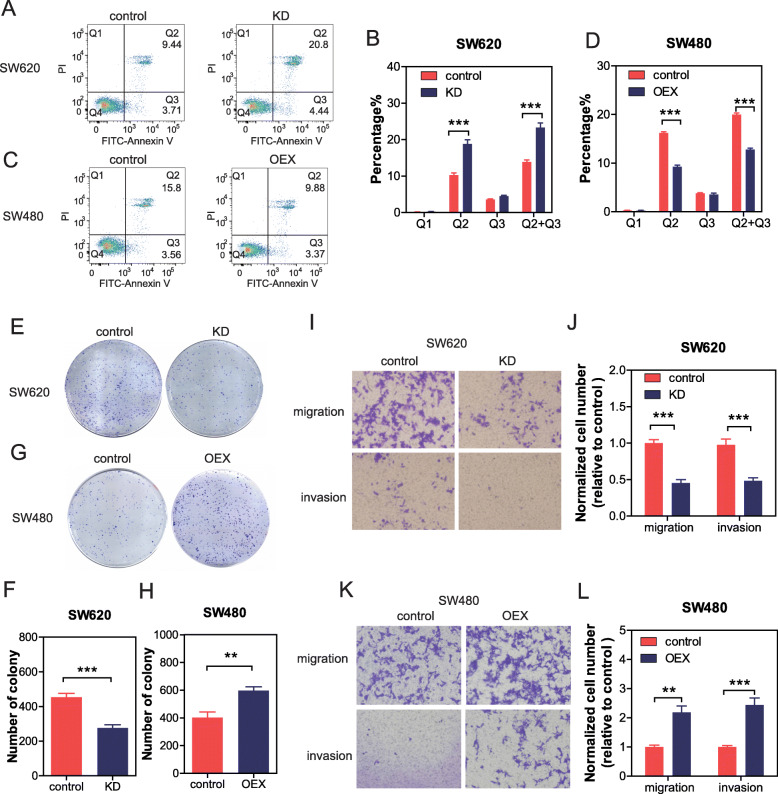
CircPTK2 promoted aggressive phenotypes of CRC cells in vitro. Cells were transfected with circPTK2 siRNA or circPTK2- overexpressing plasmid for 48 h. Then cells were harvested for annexin-V and PI staining. Cell apoptosis was detected with flow cytometry analysis. The cells were stained by crystal violet to evaluate cell proliferation, migration, and invasion capability. **a**, **b** The effect of circPTK2 knockdown on SW620 apoptosis. **c**, **d** The effect of circPTK2 overexpression on SW480 apoptosis. **e**-**h** Representative images and quantification results of the colony formation of cells. **i**-**l** Representative images and quantification results of the migration and invasion of cells. The data are the mean ± SEM. *P<0.05, **P<0.01, ***P<0.001. *P* values were calculated by one-way ANOVA

To investigate the roles of circPTK2 in malignant phenotypes, we analyzed cell apoptosis, colony formation, migration, and invasion in CRC cells. Firstly, we detected the expression levels of circPTK2 in CRC cell lines with different metastatic potential. Enhanced signals were found in SW620 cells (with high metastatic potential) than in SW480 cells (derived from the primary lesion). circPTK2 knockdown significantly decreased the apoptosis in SW620 cells (Fig. 3a, b), but not in SW480 cells (Additional file 2: Figure S2A, B). On the contrary, circPTK2 overexpression increased the apoptosis in SW480 cells (Fig. 3c, d), but not in SW620 cells (Additional file 2: Figure S2C, D). Moreover, colony formation, migration, and invasion assays showed that circPTK2 knockdown significantly inhibited the proliferative, migrant, and invasive capacity of SW620 and LOVO cells (Fig. 3e, f, i, j and Additional file 2: Figure S2E, F, I, J). These results were confirmed by circPTK2 overexpression in SW480 and HCT15 cells (low-level circPTK2) (Fig. 3g, h, k, l, and Additional file 2: Figure S2G, H, K, L).

### CircPTK2 promoted tumor growth and metastasis in vivo

**Fig. 4:**
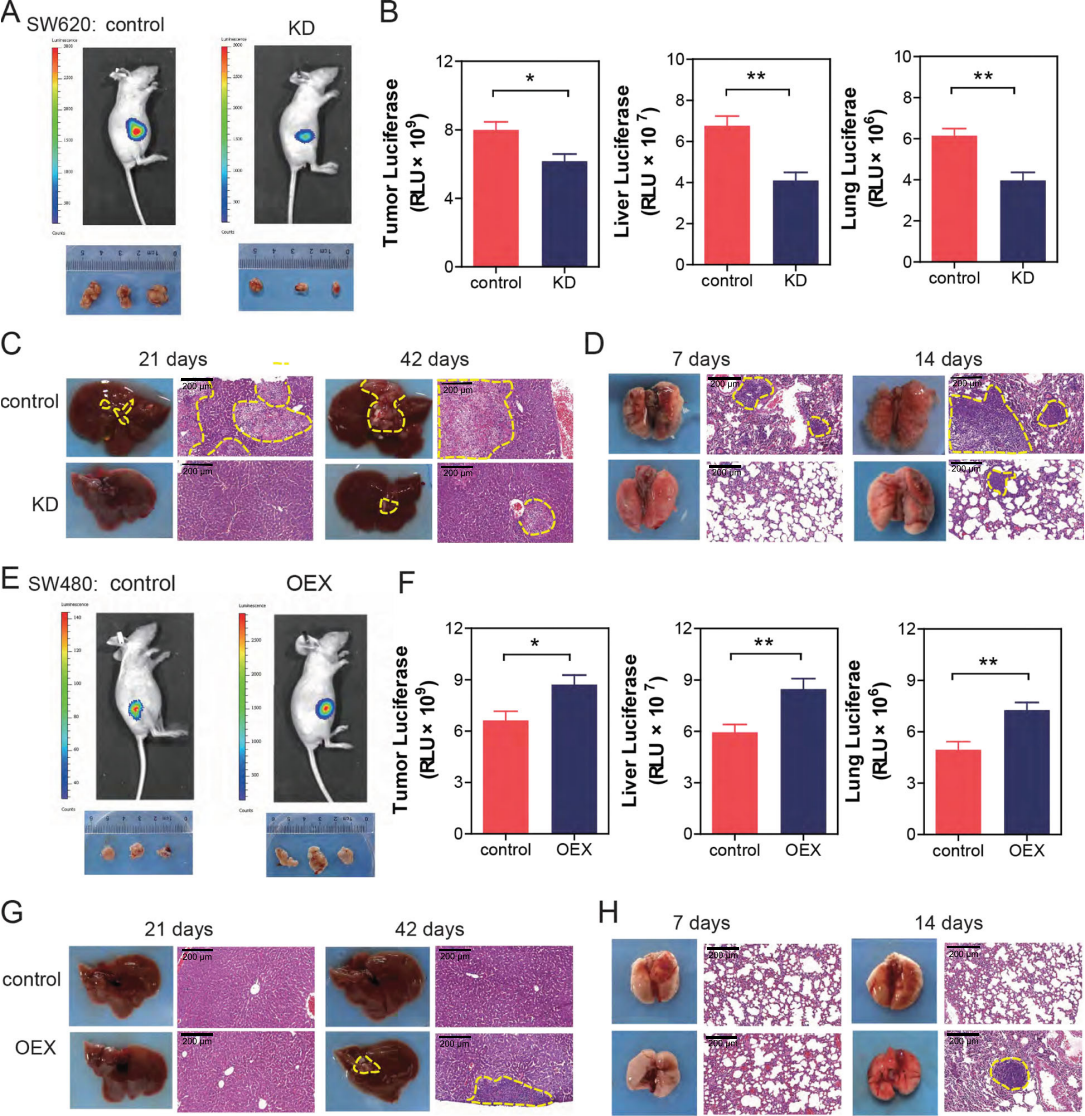
CircPTK2 promoted tumor growth and metastasis in a xenograft mouse model. SW620 or SW480 cells stably expressing luciferase were transfected with the indicated siRNAs or the indicated overexpressing-plasmid. After 72 h, cells were injected into the tail veins of nude mice (*n* = 6). **a** Representative bioluminescence images of mice bearing SW620 cells were obtained. **b** Luciferase activity of tumor, liver and lung was quantified in mice bearing SW620 cells. **c** SW620 cells, transfected with the indicated siRNAs, were injected to spleen of mice. After 21 and 42 days, livers were obtained. Representative images of liver and its HE staining were shown. **d** SW620 cells were injected into the tail veins of nude mice. After 7 and 14 days, lungs were obtained. Representative images of lung and its HE staining are shown. **e** Representative bioluminescence images of mice bearing SW480 cells were obtained. **f** Luciferase activity of tumor, liver and lung was quantified in mice bearing SW480 cells. **g** SW480 cells, transfected with the indicated overexpressing-plasmid, were injected to spleen of mice. After 21 and 42 days, livers were obtained. Representative images of liver and its HE staining are shown. **h** SW480 cells were injected into the tail veins of nude mice. After 7 and 14 days, lungs were obtained. Representative images of lung and its HE staining are shown. The data are the mean ± SEM. *P<0.05, **P<0.01, ***P<0.001. *P* values were calculated by one-way ANOVA

To evaluate the functional role of circPTK2 in CRC, a luciferase-expressing SW620 colon tumor cell line, was used to generate tumors orthotopically in female nude mice. Ex vivo imaging of organs showed a significant decrease in tumor cell burden in the primary location, liver, and lung associated with circPTK2 knockdown (Fig. 4a, b). These data were confirmed by grafting mice with luciferase-expressing LOVO cell line (Additional file 3: Figure S3A, B). To further elucidate the effect of circPTK2 in CRC metastasis, we also conducted a colon cancer hepatic and pulmonary metastasis experiment. Results showed that circPTK2 knockdown significantly blunted the spontaneous hepatic metastasis following intrasplenic injection or lung metastasis following tail vein injection of SW620, respectively (Fig. 4c, d). Together, these data suggested that circPTK2 knockdown inhibited the capacity of tumorigenesis and metastasis. Finally, overexpression assay showed that artificial accumulation of circPTK2 enhanced the capacity of tumor growth and metastasis in vivo (Fig. 4e, f, g, h, and Additional file 3: Figure S3C, D).

### CircPTK2 targeted vimentin protein to regulate the growth and metastasis of CRC

**Fig. 5:**
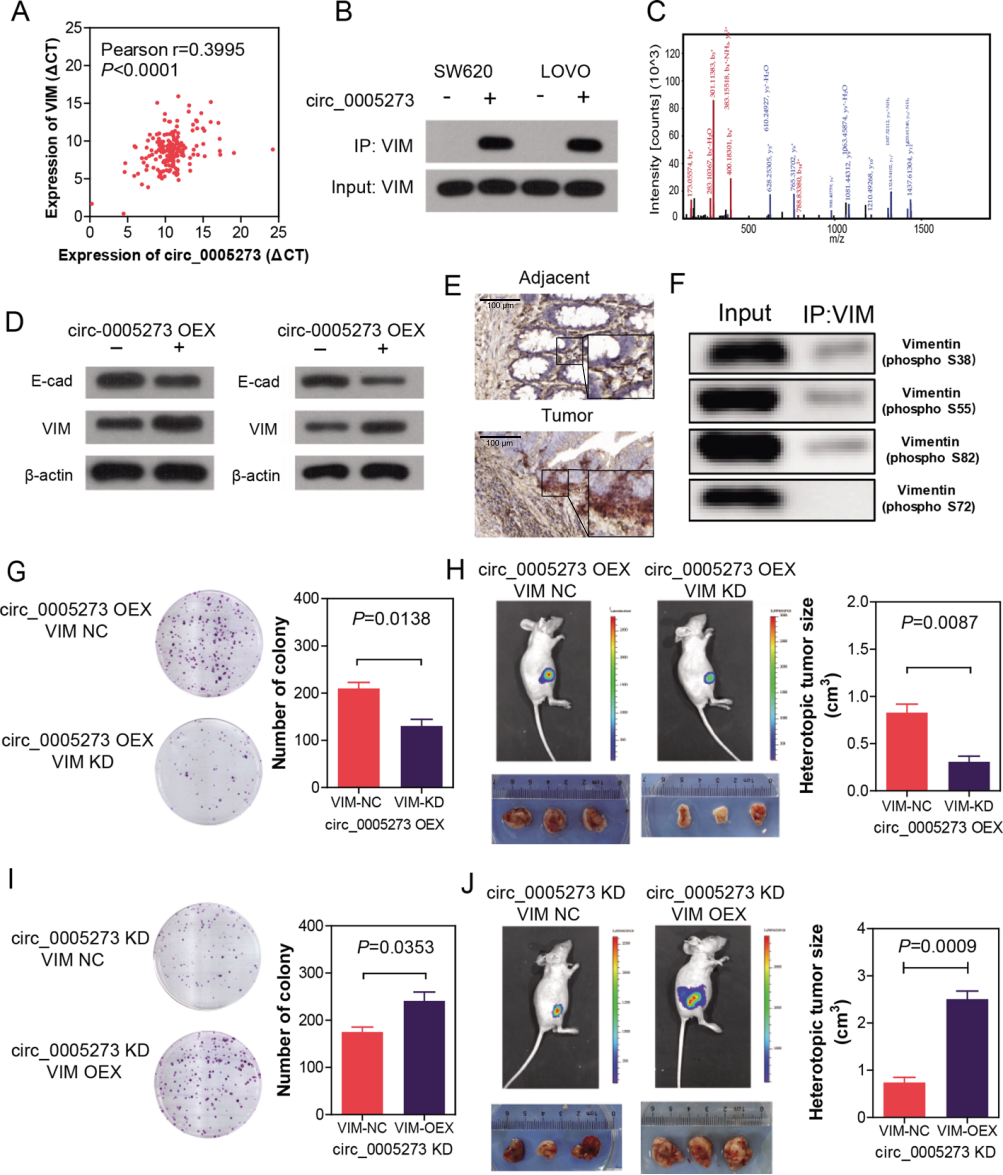
CircPTK2 targeted vimentin protein to regulate growth and metastasis of CRC. **a** The correlation between the expression level of circPTK2 and vimentin. **b** Western blot assay showing the protein vimentin pulled down by biotin-labeled circular RNA probes from the lysates of SW620 and LOVO cells. **c** Mass spectrometry assays revealing the protein vimentin pulled down by biotin-labeled circPTK2 from the lysates of SW620 and LOVO cells. **d** Western blot assay showing circPTK2 overexpression up-regulated vimentin, MMP2/9 and CXCR4, and down-regulated E-cadherin. **e** Dual RNAscope and IHC assay indicating the co-localization of circPTK2 (red) and vimentin (brown) in CRC tissues. **f** RNA pulldown and western blotting assay showing the target sites for circPTK2 on vimentin. **g**-**h** SW480 cells were transfected with circPTK2-overexpressing plasmid as well as vimentin siRNA for 48 h. Cells were used for staining with crystal violet or injected into subcutaneous tissue of nude mice. Colony formation and tumor growth were detected. Colony formation and tumor growth of SW480 cells were shown. **i**-**j** SW620 cells were transfected with circPTK2 siRNA plasmid as well as vimentin-overexpressing plasmid for 48 h. Cells were used for staining with crystal violet or injected into subcutaneous tissue of nude mice. Colony formation and tumor growth of SW620 cells were shown. *P* values were calculated by *t-*test

To investigate the mechanism by which circPTK2 regulates the tumorigenesis, growth, and metastasis of CRC, we used RNA biotin-labeled pulldown and mass spectrometry to identify the target of circPTK2. Fortunately, we found vimentin protein significantly bound with circPTK2 (Fig. 5b, c and Additional file 4: Figure S4). The expression level of circPTK2 is significantly correlated with vimentin (Fig. 5a). Dual RNAscope and IHC assay confirmed the co-localization of circPTK2 and vimentin in CRC tissues (Fig. 5e). In addition, western blotting results showed that circPTK2 overexpression markedly promoted vimentin, MMP2/9 and CXCR4 expression in SW480 and HCT15 cells, and significantly decreased the E-cadherin expression (Fig. 5d and Additional file 6: Figure S6). Furthermore, we confirmed the target sites for circPTK2 on vimentin by RNA pulldown and western blotting with antibodies against vimentin (phosphor Ser38, Ser55, Ser72, Ser82), we found circPTK2 bound to vimentin on sites Ser38, Ser55 and Ser82, rather than Ser72 (Fig. 5f).

To determine whether the binding of circPTK2 and vimentin protein functionally mediated the regulation of CRC growth and metastasis, we first stably overexpressed the circPTK2 in SW480 and HCT15 cells (low-level circPTK2), sequentially followed by knocking down vimentin expression with siRNA. Results showed that vimentin knockdown markedly blocked the effect of circPTK2 expression in colony formation and tumor cell burden (Fig. 5g, h, and Additional file 5: Figure S5A, B). Similar results were acquired in SW620 and LOVO cells (high-level circPTK2) that vimentin overexpression significantly increased colony formation and tumor cell burden, which downregulated by the knockdown of circPTK2 (Fig. 5i, j, and Additional file 5: Figure S5C, D).

### shRNA specifically targeting circPTK2 significantly inhibited tumor metastasis in a patient-derived tumor xenograft (PDTX) model of CRC

**Fig. 6:**
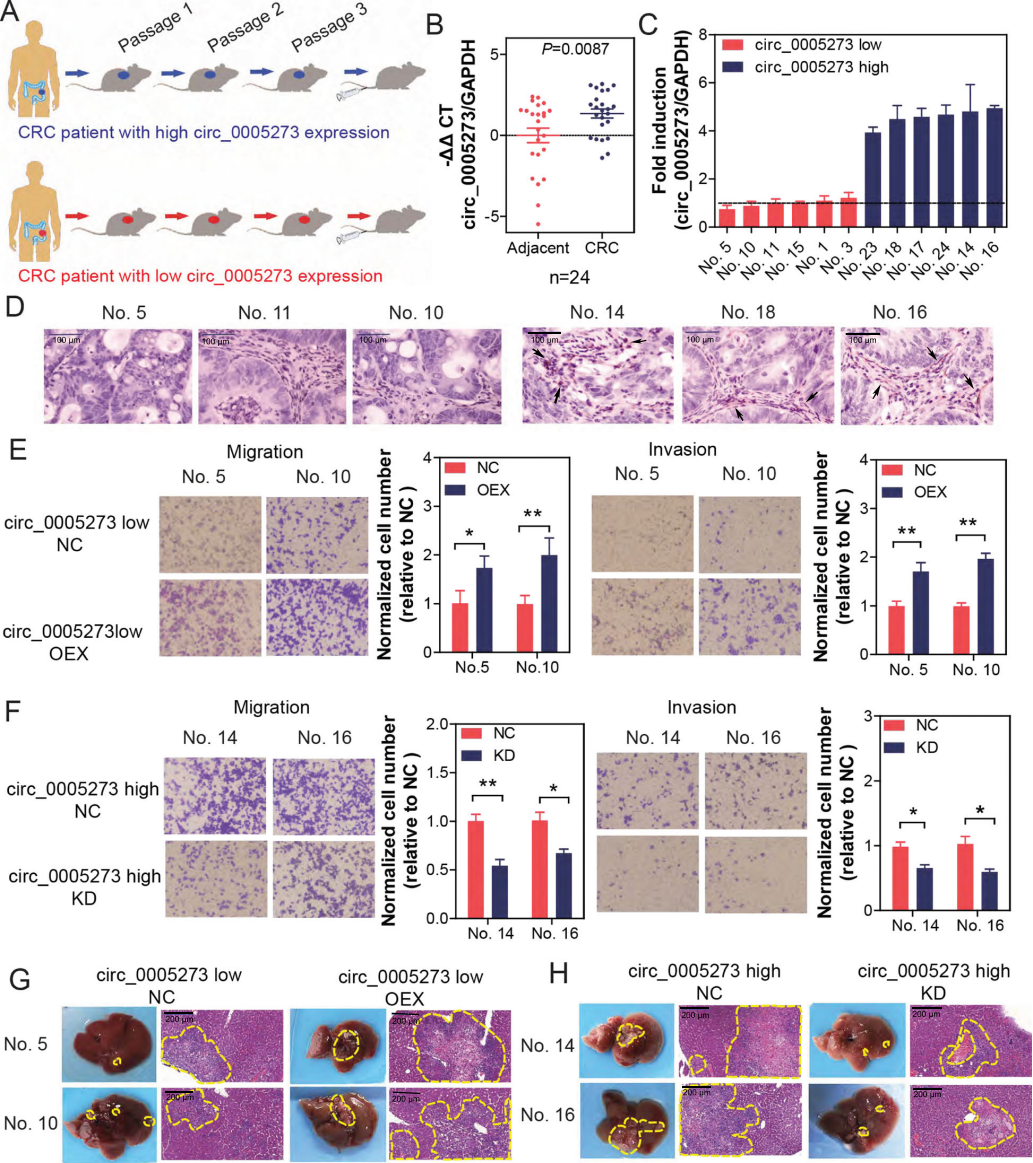
Tail vein injection of shRNA specifically targeting circPTK2 significantly inhibited tumor metastasis in a patient-derived tumor xenograft (PDTX) model of CRC. PDTX model was established by using tumor tissues of 24 phase I and II patients. **a** Schematic of tail vein injection of circPTK2 or shRNA specifically targeting circPTK2 in the PDTX model. **b** circPTK2 levels of parental adjacent and tumor CRC tissue were assessed by qRT-PCR. **c** Six highest and lowest circPTK2-expressing tissues were selected for establishment of PDTX. circPTK2 levels of orthotopic xenografts isolated from mice of PDTX were assessed by qRT-PCR. **d** Vimentin expression in the tumor tissues of PDTX was analyzed via IHC (black arrows showed the vimentin^+^ areas and cells). **e** Cells were isolated from mice bearing lower circPTK2-expressing xenografts, which were injected with vector and circPTK2-overexpressing plasmid by tail vein. Migration and invasion of cells were detected. **f** Cells were isolated from mice bearing higher circPTK2-expressing xenografts, which were injected with control siRNA and circPTK2 siRNA by tail vein. Migration and invasion of cells were detected. **g** Cells were treated as Figure 6e and then injected into spleen of nude mice. Livers were isolated and stained with HE. Representative images of livers and their HE staining are shown. **h** Cells were treated as Figure 6f and then injected into spleen of nude mice. Representative images of livers and their HE staining are shown. The data are the mean ± SEM. *P<0.05, **P<0.01. *P* values were calculated by one-way ANOVA

We established a PDTX model using primary tumor cells isolated from 24 CRC patients (Fig. 6a). Firstly, we confirmed that the expression levels of circPTK2 were equal between the original donor patients and isolated cells from the PDTX (Fig. 6b). Based on the screening, we chose six highest and six lowest circPTK2-expressing tumor tissue for the further functional assays (Fig. 6c), and we detected the expression level of vimentin in three highest and three lowest circPTK2-expressing tumor tissues by IHC. Results showed that the expression of vimentin significantly increased in high-level circPTK2 tumor tissue and obviously decreased in low-level circPTK2 tumor tissue (Fig. 6d). Subsequently, mice bearing lower circPTK2-expressing xenografts were injected with control and circPTK2-overexpressing lentivirus by tail vein, whereas mice bearing higher circPTK2-expressing xenografts were injected with control shRNA and circPTK2 shRNA lentivirus by tail vein. Results showed that the migration and invasion was decreased by circPTK2 knockdown, whereas they were increased by circPTK2 overexpression (Fig. 6e and f). Similar results were obtained in liver metastasis following intrasplenic injection of tumor cells isolated from the PDTX model, in parallel with results from injection of cell line (Fig. 6g and h).

## Discussion

In this study, we found circPTK2, a circRNA derived from PTK2 gene with unknown molecular functions, was elevated and associated with poor prognosis. In vitro and in vivo studies showed that circPTK2 possesses oncogenic capability according to promoting cell proliferation, migration, and metastasis. To the best of our knowledge, this is the first report that exhaustively investigates the expression, regulation, function, and clinical implication of circPTK2 (hsa_circ_0005273) in CRC.

Compared with other RNAs, such as miRNAs and lncRNAs, circRNAs are more suitable as potential cancer biomarkers. First, the lack of 5′ or 3′ ends makes circRNAs highly resistant to RNase activity [6, 12, 28]. Second, they often express in a tissue- and developmental-stage-specific manner; and third, they are plentiful in various tissues and body fluids including blood, plasma, serum, and even in exosomes, making them potential candidates for liquid biopsy biomarkers [29, 30]. As expected, there are already many circRNAs recognized as cancer biomarkers. CiRS-7 and circHIPK3 were respectively identified to be promising prognostic biomarkers in CRC [17, 19]. In the present study, we found that circPTK2 might act as a potential biomarker for early diagnosis of CRC metastasis.

A previous study reported that circPTK2 (hsa_circ_0003221) promotes the proliferation and migration of bladder cancer cells [31]. One recent study suggested circPTK2 (hsa_circ_0008305) inhibits TGF-β-induced epithelial- mesenchymal transition and metastasis by controlling TIF1γ in non-small cell lung cancer [32]. Together with our study, all these three circRNAs (hsa_circ_0005273, hsa_circ_0008305, and hsa_circ_0003221) were derived from the same pre-mRNA PTK2. However, they have distinctive sequences. CircRNA hsa_circ_0008305 with a spliced sequence length of 584 nt in circBase is back-spliced of seven exons (exons 8, 9, 10, 11, 12, 13 and 14) of PTK2. CircRNA hsa_circ_0005273 with a spliced sequence length of 357 nt is back-spliced of three exons (exons 27, 28, and 29). CircRNA hsa_circ_0003221 with a spliced sequence length of 625 nt is back-spliced of five exons (exons 3, 4, 5, 6 and 7). Therefore, the functions of these three circRNA may be distinguished.

CircRNAs have been reported to interact with different proteins to form specific circRNPs that subsequently influence modes of action of associated proteins [33]. For example, circANRIL was bound to the essential 60S-preribosomal assembly factor pescadillo homolog 1 (PES1) and suppressed ribosome biogenesis in vascular smooth muscle cells and macrophages, resulting in nucleolar stress and cell death, which are key cellular events in atherosclerosis [14]. CircMbl was also found to be associated with multifunctional protein MBL, which promotes the biogenesis of circMbl that is produced from the same gene locus. This circRNA could then sponge out the excess MBL protein by binding to it [34]. Our results suggesting physical binding to circPTK2 to vimentin, which is distinguished from circPTK2 (hsa_circ_0008305), acting as a sponge for miR-429 / miR-200b-3p in non-small cell lung cancer cells and hereby inhibiting EMT in lung cancer cells [32]. Recently, some circRNAs with tumor suppressive properties have been found to play important roles in tumorigenesis and metastasis, and can be used as therapeutic targets for cancer. CircITGA7 was found to inhibit the proliferation and metastasis of CRC cells by inhibiting the Ras signaling pathway and promoting ITGA7 transcription. Furthermore, hsa_circ_0014717 suppressed CRC growth by upregulating P16 expression [35]. These indicate that circRNAs are gradually becoming carcinogenic stimulators or tumor suppressors in cancer.

Vimentin is a type 3 intermediate filament protein that is an critical marker of mesenchymal cells in EMT. It is responsible for maintaining cell shape and stabilizing cytoskeletal interactions. Vimentin serves as an organizer of many key proteins involved in cell attachment and migration [36]. Vimentin is a potential cancer therapeutic target, since it is overexpressed in a number of cancers, and influences cell shape and motility in the process of EMT that occurs during metastasis [37, 38]. Current studies have shown that phosphorylation of vimentin serine residues inhibits subunit polymerization, then promoting the decomposition of vimentin filaments and increasing the solubility of the protein [39]. For example, Protein kinase A (PKA) phosphorylates vimentin mainly at Ser38 and Ser72 sites, resulting in decreased filament formation in vivo, which indicates that phosphorylation mainly regulates the decomposition of vimentin intermediate filaments [40]. During mitosis, CDK1 phosphorylated vimentin at Ser55. This phosphorylation provides a PLK binding site for vimentin-PLK interaction. PLK further phosphorylated vimentin at Ser82, which may be a memory phosphorylation site and lead to the marked disassembly of vimentin filaments [41, 42]. We found that circPTK2 (hsa_circ_0005273) promoted expression of vimentin via physically binding to its phosphorylation sites Ser38, Ser55 and Ser82.

Limitation of our study should be mentioned. It remains unclear that the regulatory mechanism of circPTK2 to promote CRC growth, and whether any miRNAs or other transcription factors are involved in the regulation of circPTK2 to metastatic process of CRC. CircPTK2 plays a part in tumor progression and metastasis. Therefore, how circPTK2 regulates CRC progression and metastasis warrants further investigation.

## Conclusions

In summary, we reported that increased expression levels of hsa_circ_0005273, spliced from the pre-mRNA PTK2, was involved in the metastasis of colorectal cancer via physical binding to phosphorylation sites Ser38, Ser55 and Ser82 of vimentin. Our study provides an exploitable therapeutic target for CRC patients, despite that the underlying mechanisms need further investigation.

### Supplementary information

**Additional file 1: Figure S1.** The circPTK2 is elevated in serum of CRC patients. (A) Levels of circPTK2 in serum from healthy controls (NOR) and CRC patients (CRC) in the testing, validation, and the combination set were detected by qPCR. (B) The abundance of circPTK2 in CRC patients with different clinical characteristics were further analyzed. Data are presented as -ΔΔCt by one-way ANOVA with three technical replicates each. (C) Receiver operating characteristic (ROC) analysis was used to evaluate the diagnosis value of circPTK2 in CRC with different clinical characteristics. T: primary tumor; N: node metastasis; M: distant metastasis. Shown are the calculated ROC curves.

**Additional file 2: Figure S2.** CircPTK2 promoted aggressive phenotypes of CRC cells in vitro. Cells were transfected with circPTK2 siRNA or circPTK2-overexpressing plasmid for 48 h. Then cells were harvested for annexin-V and PI staining. Cell apoptosis was detected with flow cytometry analysis. The cells were stained by crystal violet to evaluate cell proliferation, migration, and invasion capability. (A, B) The effect of circPTK2 knockdown on SW480 apoptosis. (C, D) The effect of circPTK2 overexpression on SW620 apoptosis. (E-H) Representative images and quantification results of the colony formation of cells. (I-L) Representative images and quantification results of the migration and invasion of cells. The data are the mean ± SEM. *P<0.05, **P<0.01, ***P<0.001. *P* values were calculated by one-way ANOVA.

**Additional file 3: Figure S3.** CircPTK2 promoted tumor growth in a xenograft mouse model. LOVO cells stably expressing luciferase were transfected with the indicated siRNAs and HCT15 cells stably expressing luciferase were transfected with the indicated overexpressing-plasmid. After 72 h, cells were injected into the tail veins of nude mice (*n* = 6). (A) Representative bioluminescence images of mice bearing LOVO cells were obtained. (B) Luciferase activity of tumor, liver, and lung was quantified in mice bearing LOVO cells. (C) Representative bioluminescence images of mice bearing HCT15 cells were obtained. (D) Luciferase activity of tumor, liver and lung was quantified in mice bearing HCT15 cells. The data are the mean ± SEM. *P<0.05, **P<0.01, ***P<0.001. *P* values were calculated by one-way ANOVA.

**Additional file 4:** **Figure S4.** The mass spectrometry of vimentin. Each mass spectrum represents a peptide. The protein binding to circPTK2 was vimentin.

**Additional file 5: Figure S5.** CircPTK2 targeted vimentin protein to regulate growth and metastasis of CRC. (A-B) HCT15 cells were transfected with circPTK2-overexpressing plasmid as well as vimentin siRNA for 48 h. Cells were used for staining with crystal violet or injected into subcutaneous tissue of nude mice. Colony formation and tumor growth were detected. Colony formation and tumor growth of HCT15 cells were shown. (C-D) LOVO cells were transfected with circPTK2 siRNA plasmid as well as vimentin-overexpressing plasmid for 48 h. Cells were used for staining with crystal violet or injected into subcutaneous tissue of nude mice. Colony formation and tumor growth of LOVO cells were shown. *P* values were calculated by t test.

**Additional file 6: Figure S6.** CircPTK2 promoted MMP2/9 and CXCR4 expression in CRC cells. SW480 and HCT15 cells were transfected with circPTK2-overexpressing plasmid for 48 h, western blot assay showing circPTK2 overexpression up-regulated MMP2/9 and CXCR4.

**Additional file 7: Table S1.** Demographic information of colorectal cancer (CRC) and healthy control for PCR analysis.

**Additional file 8: Table S2.** Demographic information of colorectal cancer (CRC) patients subjected to TMA.

**Additional file 9: Table S3.** The detailed information of patients subjected to circRNA microarray analysis.

**Additional file 10.** Supplementary methods.

## Data Availability

The data used or analyzed during this study are included in this article and available from the corresponding author upon reasonable request.

## Abbreviations

circRNAs: circular RNAs
CRC: Colorectal cancer
EMT: Epithelial–mesenchymal transition
PDTX: Patient-derived tumor xenograft
TMA: Tissue microarray
