# Correction: CircPTK2 (hsa_circ_0005273) as a novel therapeutic target for metastatic colorectal cancer

**DOI:** 10.1186/s12943-024-01983-3

**Published:** 2024-04-02

**Authors:** Hongbao Yang, Xiaobo Li, Qingtao Meng, Hao Sun, Shenshen Wu, Weiwei Hu, Guilai Liu, Xianjing Li, Yong Yang, Rui Chen

**Affiliations:** 1 State Key Laboratory of Natural Medicines, Institute of Pharmaceutical Science, China Pharmaceutical University, Nanjing, 211198 China; 2 Key Laboratory of Environmental Medicine Engineering, Ministry of Education, School of Public Health, Southeast University, Nanjing, 210009 China; 3 School of Public Health, Advanced Innovation Center for Human Brain Protection, Capital Medical University, Beijing, 100069 People’s Republic of China; 4 Beijing Key Laboratory of Environmental Toxicology, Capital Medical University, Beijing, 100069 People’s Republic of China; 5 School of Pharmacy, Xuzhou Medical University, 209 Tongshan Road, Xuzhou, 221004 Jiangsu China

**Correction: Mol Cancer 19, 13 (2020)**

10.1186/s12943-020-1139-3

The authors are writing to request correction to the following paper published in Molecular Cancer [1].

They found Fig. 4D appeared incorrectly, as errors were introduced during preparation of these figures. They thus replace Fig. 4D at 7 days with the correct image, as follow:

Corrected Fig. 4D:


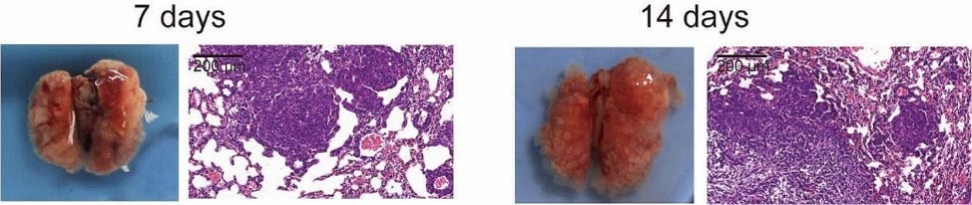


In addition, the sequence of primers for circPTK2 should be corrected as follow:

5’- AGAGGAAAGATTTCTGCCCA-3’(forward) and 5’-ATTCCATGTGAACCAGGGTA-3’(reverse).
