# Supplementary material for: CircPTK2 (hsa_circ_0005273) as a novel therapeutic target for metastatic colorectal cancer
Source: Mol Cancer. 2020 Jan 23;19:13. doi: 10.1186/s12943-020-1139-3 (PMC6977296; doi:10.1186/s12943-020-1139-3)
Supplement: Supplementary file 1 — Additional file 1: Figure S1. The circPTK2 is elevated in serum of CRC patients. (A) Levels of circPTK2 in serum from healthy controls (NOR) and CRC patients (CRC) in the testing, validation, and the combination set were detected by qPCR. (B) The abundance of circPTK2 in CRC patients with different clinical characteristics were further analyzed. Data are presented as -ΔΔCt by one-way ANOVA with three technical replicates each. (C) Receiver operating characteristic (ROC) analysis was used to evaluate the diagnosis value of circPTK2 in CRC with different clinical characteristics. T: primary tumor; N: node metastasis; M: distant metastasis. Shown are the calculated ROC curves. [file 12943_2020_1139_MOESM1_ESM.docx]

**Additional file 1**

**Supplementary Figure 1**


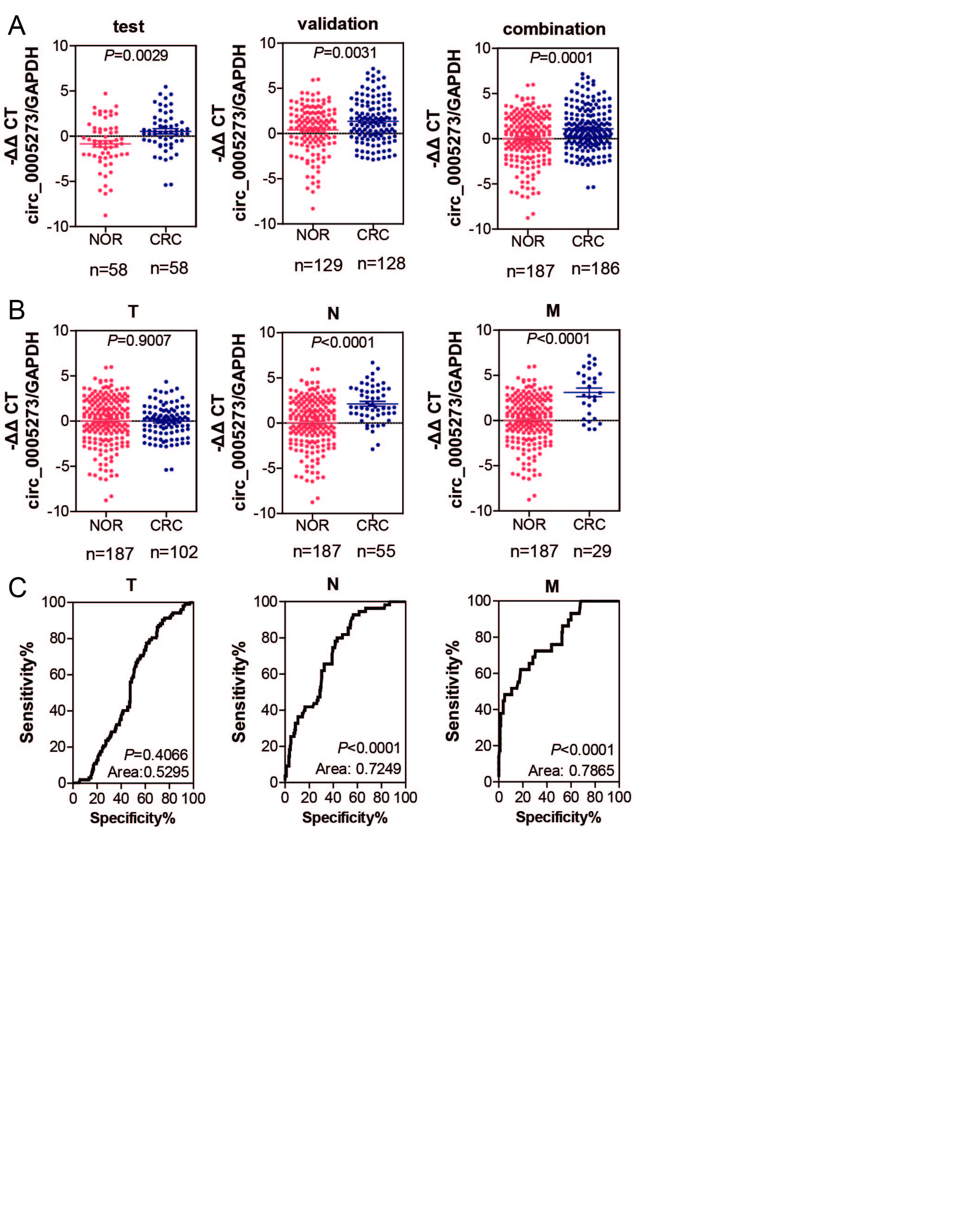


**Supplementary Figure 1. The circPTK2 is elevated in serum of CRC patients.**

1. Levels of circPTK2 in serum from healthy controls (NOR) and CRC patients (CRC) in the testing, validation, and the combination set were detected by qPCR. (B) The abundance of circPTK2 in CRC patients with different clinical characteristics were further analyzed. Data are presented as -ΔΔCt by one-way ANOVA with three technical replicates each. (C) Receiver operating characteristic (ROC) analysis was used to evaluate the diagnosis value of circPTK2 in CRC with different clinical characteristics. T: primary tumor; N: node metastasis; M: distant metastasis. Shown are the calculated ROC curves.
