# Supplementary material for: CircPTK2 (hsa_circ_0005273) as a novel therapeutic target for metastatic colorectal cancer
Source: Mol Cancer. 2020 Jan 23;19:13. doi: 10.1186/s12943-020-1139-3 (PMC6977296; doi:10.1186/s12943-020-1139-3)
Supplement: Supplementary file 2 — Additional file 2: Figure S2. CircPTK2 promoted aggressive phenotypes of CRC cells in vitro. Cells were transfected with circPTK2 siRNA or circPTK2-overexpressing plasmid for 48 h. Then cells were harvested for annexin-V and PI staining. Cell apoptosis was detected with flow cytometry analysis. The cells were stained by crystal violet to evaluate cell proliferation, migration, and invasion capability. (A, B) The effect of circPTK2 knockdown on SW480 apoptosis. (C, D) The effect of circPTK2 overexpression on SW620 apoptosis. (E-H) Representative images and quantification results of the colony formation of cells. (I-L) Representative images and quantification results of the migration and invasion of cells. The data are the mean ± SEM. *P<0.05, **P<0.01, ***P<0.001. P values were calculated by one-way ANOVA. [file 12943_2020_1139_MOESM2_ESM.docx]

**Additional file 2**

**Supplementary Figure 2**


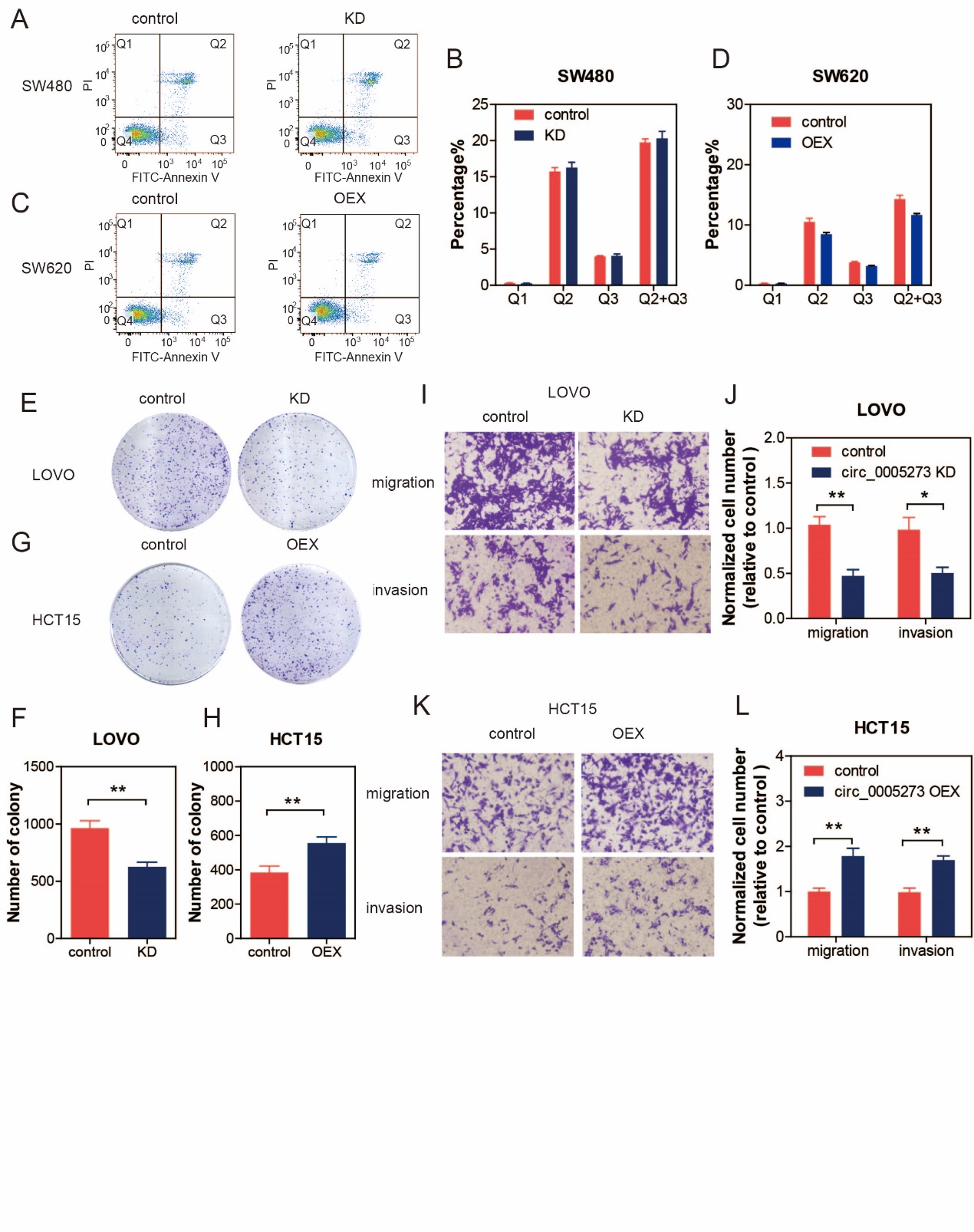


**Supplementary Figure 2. CircPTK2 promoted aggressive phenotypes of CRC cells in vitro.** Cells were transfected with circPTK2 siRNA or circPTK2-overexpressing plasmid for 48 hours. Then cells were harvested for annexin-V and PI staining. Cell apoptosis was detected with flow cytometry analysis. The cells were stained by crystal violet to evaluate cell proliferation, migration, and invasion capability. (A, B) The effect of circPTK2 knockdown on SW480 apoptosis. (C, D) The effect of circPTK2 overexpression on SW620 apoptosis. (E-H) Representative images and quantification results of the colony formation of cells. (I-L) Representative images and quantification results of the migration and invasion of cells. The data are the mean ± SEM. **P*＜0.05, ***P*＜0.01, ****P*＜0.001. *P* values were calculated by one-way ANOVA.
