# Supplementary material for: CircPTK2 (hsa_circ_0005273) as a novel therapeutic target for metastatic colorectal cancer
Source: Mol Cancer. 2020 Jan 23;19:13. doi: 10.1186/s12943-020-1139-3 (PMC6977296; doi:10.1186/s12943-020-1139-3)
Supplement: Supplementary file 3 — Additional file 3: Figure S3. CircPTK2 promoted tumor growth in a xenograft mouse model. LOVO cells stably expressing luciferase were transfected with the indicated siRNAs and HCT15 cells stably expressing luciferase were transfected with the indicated overexpressing-plasmid. After 72 h, cells were injected into the tail veins of nude mice (n = 6). (A) Representative bioluminescence images of mice bearing LOVO cells were obtained. (B) Luciferase activity of tumor, liver, and lung was quantified in mice bearing LOVO cells. (C) Representative bioluminescence images of mice bearing HCT15 cells were obtained. (D) Luciferase activity of tumor, liver and lung was quantified in mice bearing HCT15 cells. The data are the mean ± SEM. *P<0.05, **P<0.01, ***P<0.001. P values were calculated by one-way ANOVA. [file 12943_2020_1139_MOESM3_ESM.docx]

**Additional file 3**

**Supplementary Figure 3**


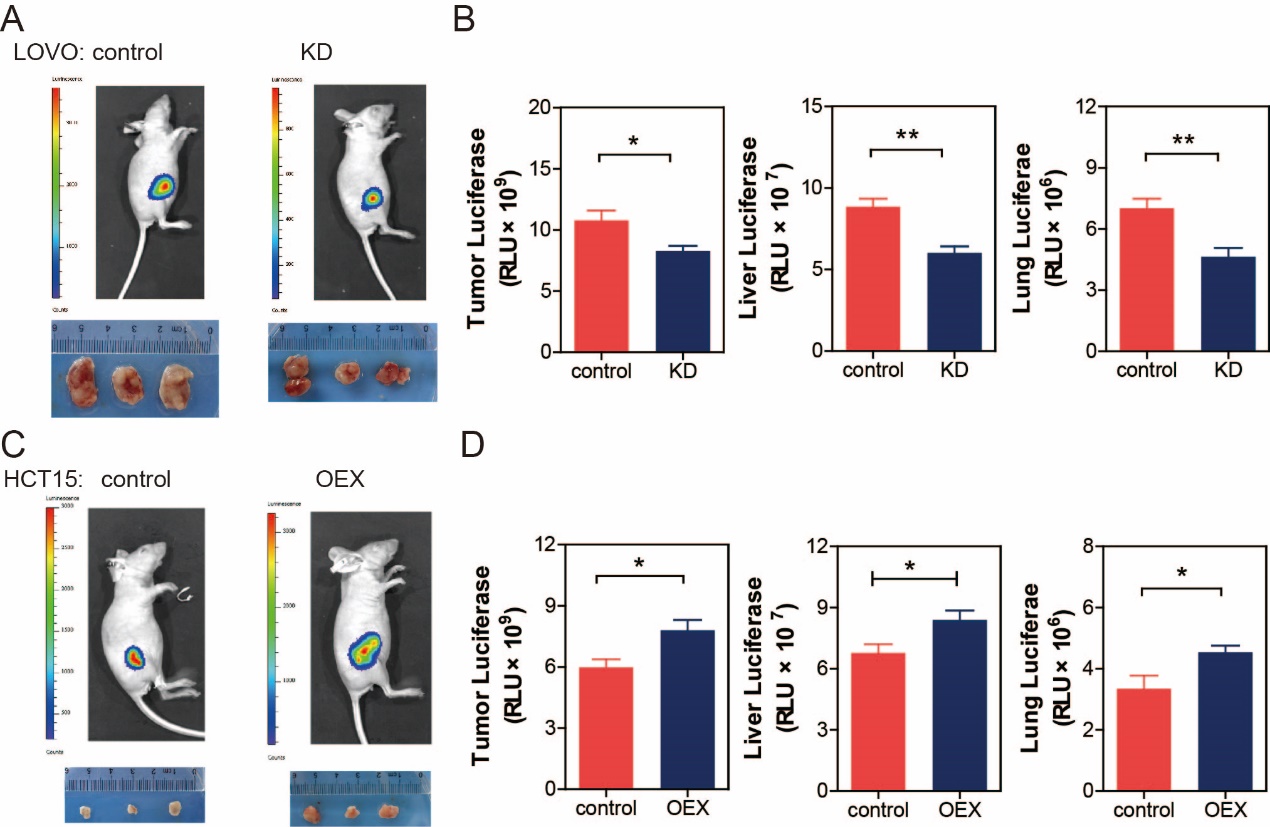


**Supplementary Figure 3. CircPTK2 promoted tumor growth in a xenograft mouse model.**

LOVO cells stably expressing luciferase were transfected with the indicated siRNAs and HCT15 cells stably expressing luciferase were transfected with the indicated overexpressing-plasmid. After 72 hours, cells were injected into the tail veins of nude mice (n=6). (A) Representative bioluminescence images of mice bearing LOVO cells were obtained. (B) Luciferase activity of tumor, liver, and lung was quantified in mice bearing LOVO cells. (C) Representative bioluminescence images of mice bearing HCT15 cells were obtained. (D) Luciferase activity of tumor, liver and lung was quantified in mice bearing HCT15 cells. The data are the mean ± SEM. **P*＜0.05, ***P*＜0.01, ****P*＜0.001. *P* values were calculated by one-way ANOVA.
