# Supplementary material for: CircPTK2 (hsa_circ_0005273) as a novel therapeutic target for metastatic colorectal cancer
Source: Mol Cancer. 2020 Jan 23;19:13. doi: 10.1186/s12943-020-1139-3 (PMC6977296; doi:10.1186/s12943-020-1139-3)
Supplement: Supplementary file 4 — Additional file 4: Figure S4. The mass spectrometry of vimentin. Each mass spectrum represents a peptide. The protein binding to circPTK2 was vimentin. [file 12943_2020_1139_MOESM4_ESM.docx]

**Additional file 4**

**Supplementary Figure 4**


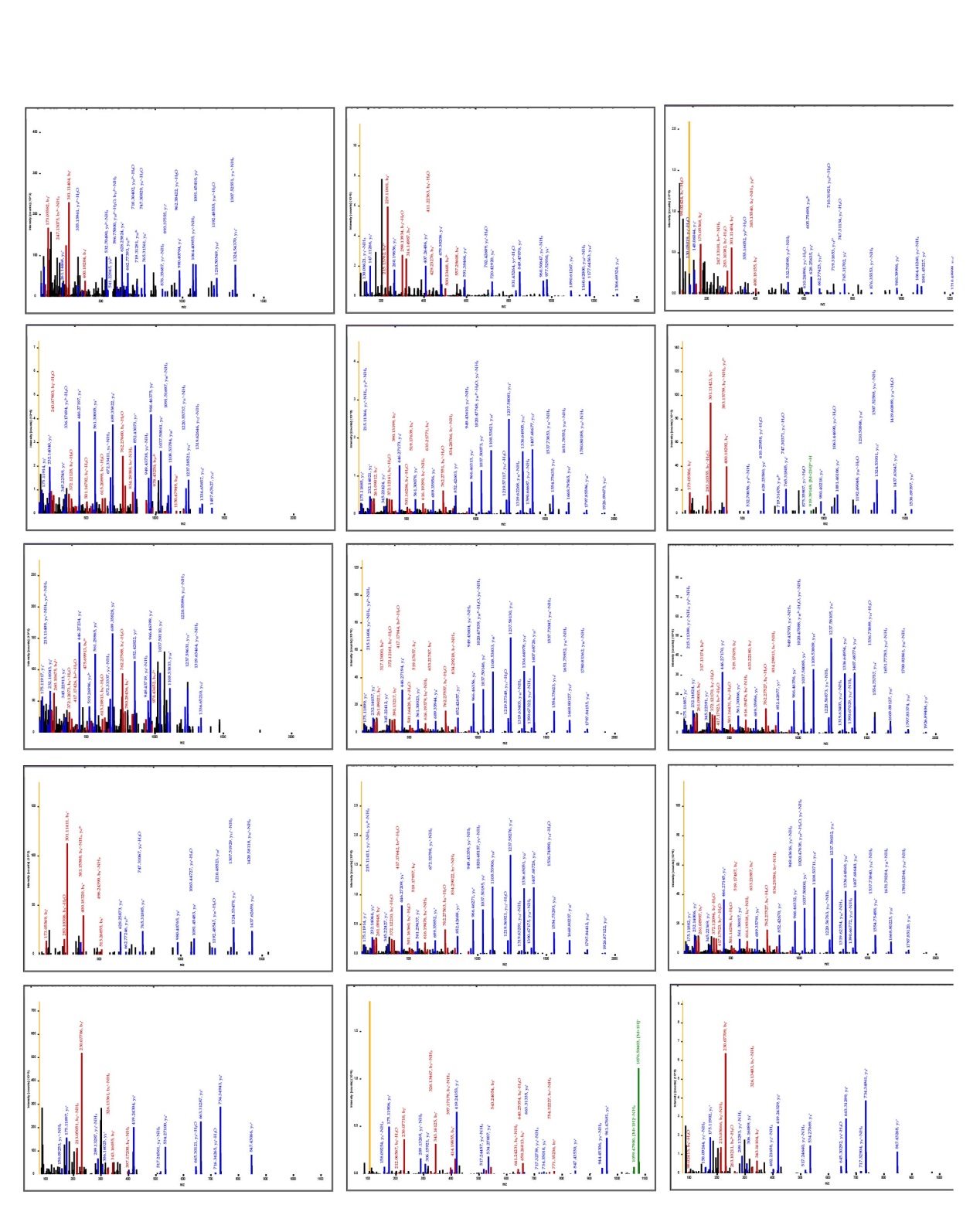


**Supplementary Figure 4. The mass spectrometry of vimentin.**

Each mass spectrum represents a peptide. The protein binding to circPTK2 was vimentin.
