# Supplementary material for: CircPTK2 (hsa_circ_0005273) as a novel therapeutic target for metastatic colorectal cancer
Source: Mol Cancer. 2020 Jan 23;19:13. doi: 10.1186/s12943-020-1139-3 (PMC6977296; doi:10.1186/s12943-020-1139-3)
Supplement: Supplementary file 5 — Additional file 5: Figure S5. CircPTK2 targeted vimentin protein to regulate growth and metastasis of CRC. (A-B) HCT15 cells were transfected with circPTK2-overexpressing plasmid as well as vimentin siRNA for 48 h. Cells were used for staining with crystal violet or injected into subcutaneous tissue of nude mice. Colony formation and tumor growth were detected. Colony formation and tumor growth of HCT15 cells were shown. (C-D) LOVO cells were transfected with circPTK2 siRNA plasmid as well as vimentin-overexpressing plasmid for 48 h. Cells were used for staining with crystal violet or injected into subcutaneous tissue of nude mice. Colony formation and tumor growth of LOVO cells were shown. P values were calculated by t test. [file 12943_2020_1139_MOESM5_ESM.docx]

**Additional file 5**

**Supplementary Figure 5**


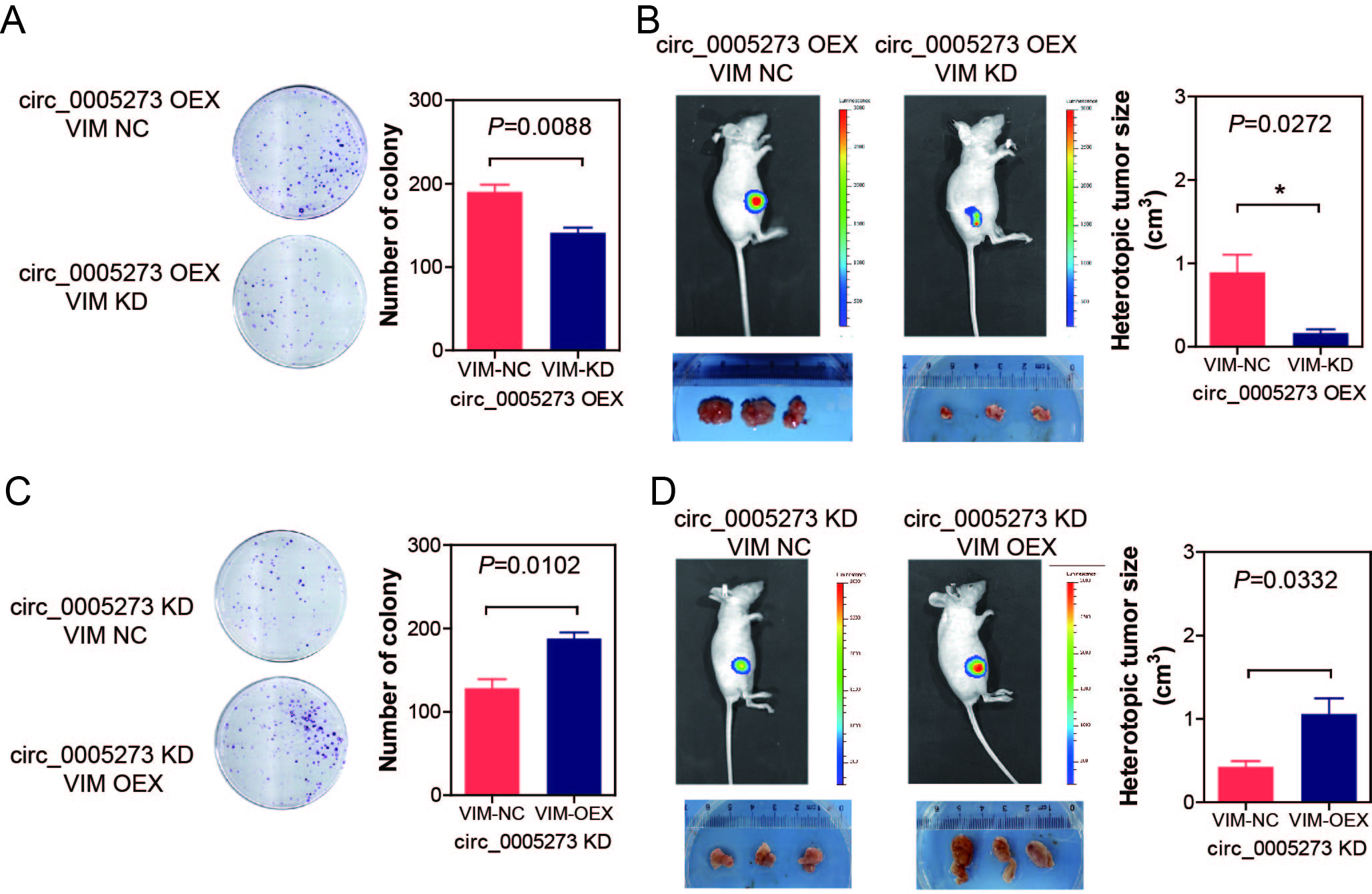


**Supplementary Figure 5. CircPTK2 targeted vimentin protein to regulate growth and metastasis of CRC.**

(A-B) HCT15 cells were transfected with circPTK2-overexpressing plasmid as well as vimentin siRNA for 48 hours. Cells were used for staining with crystal violet or injected into subcutaneous tissue of nude mice. Colony formation and tumor growth were detected. Colony formation and tumor growth of HCT15 cells were shown. (C-D) LOVO cells were transfected with circPTK2 siRNA plasmid as well as vimentin-overexpressing plasmid for 48h. Cells were used for staining with crystal violet or injected into subcutaneous tissue of nude mice. Colony formation and tumor growth of LOVO cells were shown. P values were calculated by t test.
