# Supplementary material for: CircPTK2 (hsa_circ_0005273) as a novel therapeutic target for metastatic colorectal cancer
Source: Mol Cancer. 2020 Jan 23;19:13. doi: 10.1186/s12943-020-1139-3 (PMC6977296; doi:10.1186/s12943-020-1139-3)
Supplement: Supplementary file 6 — Additional file 6: Figure S6. CircPTK2 promoted MMP2/9 and CXCR4 expression in CRC cells. SW480 and HCT15 cells were transfected with circPTK2-overexpressing plasmid for 48 h, western blot assay showing circPTK2 overexpression up-regulated MMP2/9 and CXCR4. [file 12943_2020_1139_MOESM6_ESM.docx]

**Additional file 6**

**Supplementary Figure 6**


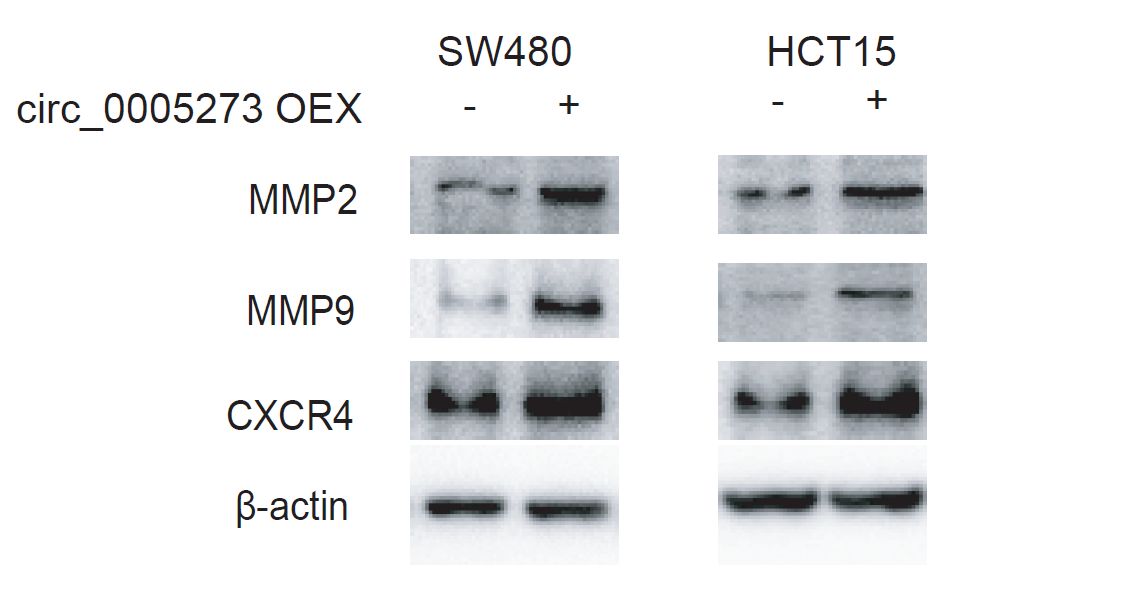


**Supplementary Figure 6. CircPTK2 promoted MMP2/9 and CXCR4 expression in CRC cells**

SW480 and HCT15 cells were transfected with circPTK2-overexpressing plasmid for 48h, western blot assay showing circPTK2 overexpression up-regulated MMP2/9 and CXCR4.
