# Supplementary material for: CircPTK2 (hsa_circ_0005273) as a novel therapeutic target for metastatic colorectal cancer
Source: Mol Cancer. 2020 Jan 23;19:13. doi: 10.1186/s12943-020-1139-3 (PMC6977296; doi:10.1186/s12943-020-1139-3)
Supplement: Supplementary file 7 — Additional file 7: Table S1. Demographic information of colorectal cancer (CRC) and healthy control for PCR analysis. [file 12943_2020_1139_MOESM7_ESM.docx]

**Additional file 7**

**Supplementary Table 1.** **Demographic information of colorectal cancer (CRC) and healthy control for PCR analysis.**

| Variables | Testing set (n=61) | |  | Validation set (n=131) | |
| --- | --- | --- | --- | --- | --- |
|  | CRC (n%) | Healthy  control (n%) |  | CRC (n%) | Healthy  control (n%) |
| Age |  |  |  |  |  |
| ≤61 | 31 (50.8) | 31 (50.8) |  | 81 (61.8) | 82 (62.6) |
| >61 | 30 (49.2) | 30 (49.2) |  | 50 (38.2) | 49 (37.4) |
| Gender |  |  |  |  |  |
| Male | 26 (42.6) | 29 (47.5) |  | 67 (51.2) | 65 (49.6) |
| Female | 35 (57.4) | 32 (52.5) |  | 64 (48.9) | 66 (50.4) |
| Location |  |  |  |  |  |
| Colon | 17 (27.9) | -- |  | 68 (51.9) | -- |
| Rectum | 44 (72.1) | -- |  | 63 (48.1) | -- |
| Grade |  |  |  |  |  |
| Low | 8 (13.1) | -- |  | 50 (38.2) | -- |
| High | 53 (86.9) | -- |  | 81 (61.8) | -- |
| TNM |  |  |  |  |  |
| I | 13 (21.3) | -- |  | 28 (21.4) | -- |
| II | 23 (37.7) | -- |  | 40 (30.5) | -- |
| III | 15 (24.6) | -- |  | 43 (32.8) | -- |
| IV | 10 (16.4) | -- |  | 20 (15.3) | -- |
