# Supplementary material for: CircPTK2 (hsa_circ_0005273) as a novel therapeutic target for metastatic colorectal cancer
Source: Mol Cancer. 2020 Jan 23;19:13. doi: 10.1186/s12943-020-1139-3 (PMC6977296; doi:10.1186/s12943-020-1139-3)
Supplement: Supplementary file 8 — Additional file 8: Table S2. Demographic information of colorectal cancer (CRC) patients subjected to TMA. [file 12943_2020_1139_MOESM8_ESM.docx]

**Additional file 8**

**Supplementary Table 2.** **Demographic information of colorectal cancer (CRC) patients subjected to TMA[**[**1**](#_ENREF_1)**].**

| Variables | CRC patient（N=1078） | |
| --- | --- | --- |
|  | n | % |
| Age |  |  |
| ≤56 | 567 | 52.6 |
| ＞56 | 511 | 47.4 |
| Gender |  |  |
| Male | 659 | 61.1 |
| Female | 419 | 38.9 |
| Location |  |  |
| Colon | 475 | 44.1 |
| Rectum | 603 | 55.9 |
| Grade |  |  |
| Low | 356 | 33 |
| Intermediate/High | 722 | 67 |
| Depth of invasion | |  |
| T1 | 35 | 3.3 |
| T2 | 244 | 22.6 |
| T3 | 204 | 18.9 |
| T4 | 595 | 55.2 |
| Lymph node metastasis | |  |
| N0 | 621 | 57.6 |
| N1 | 457 | 42.4 |
| Distant metastasis | |  |
| M0 | 958 | 88.9 |
| M1 | 120 | 11.1 |
| TNM |  |  |
| I | 196 | 18.2 |
| II | 401 | 37.2 |
| III | 361 | 33.5 |
| IV | 120 | 11.1 |

**Reference**

1. Wu S, Meng Q, Zhang C, Sun H, Lu R, Gao N, Yang H, Li X, Aschner M, Chen R: **DR4 mediates the progression, invasion, metastasis and survival of colorectal cancer through the Sp1/NF1 switch axis on genomic locus.** *Int J Cancer* 2018, **143:**289-297.
