# Supplementary material for: CircPTK2 (hsa_circ_0005273) as a novel therapeutic target for metastatic colorectal cancer
Source: Mol Cancer. 2020 Jan 23;19:13. doi: 10.1186/s12943-020-1139-3 (PMC6977296; doi:10.1186/s12943-020-1139-3)
Supplement: Supplementary file 9 — Additional file 9: Table S3. The detailed information of patients subjected to circRNA microarray analysis. [file 12943_2020_1139_MOESM9_ESM.docx]

**Additional file 9**

**Supplementary Table 3.** **The detailed information of patients subjected to circRNA microarray analysis.**

| ID | Gender | Age | Diagnosis | Stage |
| --- | --- | --- | --- | --- |
| 1 | Male | 68 | CRC | I |
| 2 | Male | 73 | CRC | I |
| 3 | Female | 68 | CRC | I |
| 4 | Male | 62 | CRC | II |
| 5 | Female | 60 | CRC | 0 |
| 6 | Female | 59 | CRC | II |
| 7 | Female | 64 | ADE | -- |
| 8 | Male | 60 | ADE | -- |
| 9 | Male | 54 | ADE | -- |
| 10 | Male | 59 | ADE | -- |
| 11 | Male | 65 | ADE | -- |
| 12 | Female | 52 | ADE | -- |
