# Supplementary material for: CircPTK2 (hsa_circ_0005273) as a novel therapeutic target for metastatic colorectal cancer
Source: Mol Cancer. 2020 Jan 23;19:13. doi: 10.1186/s12943-020-1139-3 (PMC6977296; doi:10.1186/s12943-020-1139-3)
Supplement: Supplementary file 10 — Additional file 10. Supplementary methods. [file 12943_2020_1139_MOESM10_ESM.docx]

**Additional file 10**

**Supplementary document**

**CircRNA Microarray**

Six pairs of CRC tissues (3 colon and 3 rectal carcinomas) and the corresponding adjacent non-tumor tissues, as well as six adenomas (all tubulovillous adenomas) were utilized for circRNA microarrays. The specimens were obtained from patients undergoing surgery in the Jiangsu Tumor Hospital in 2015; detailed information is shown in Supplementary Table 3.

Total RNA was isolated from tissues using TRIzol Reagent (Invitrogen, Carlsbad, CA) according to the manufacturer’s instructions. The circRNA microarray was performed using the Arraystar Human circRNA Array (8x15K, Arraystar) by a contract service at the Shanghai Kangchen Technology.

Raw data were subjected to background subtraction and normalization with the limma R-package. Discriminant circRNAs and differences between groups were analyzed using Bayes moderated t test (limma) with Benjamini Hochberg false discovery rate (FDR) at P < 0.05, unless otherwise specified. A two-fold change cut off was applied to select the up- and down-regulated circRNAs. Normalized and raw expression data were deposited in the Gene Expression Omnibus at the National Center for Biotechnology Information.

**Tissue/serum preparation and RNA isolation**

Following removal, all tissues were snap-frozen in liquid nitrogen and stored at -80°C until RNA extraction. Serum was separated from the venous blood within 1 hour by centrifugation at 3000 g for 10 minutes, followed by a 15-minute high-speed centrifugation at 12,000 g to completely remove the cell debris. The supernatant serum was collected and stored at -80℃ until use. The total RNA of tissues and serum was extracted using Trizol reagent (Invitrogen, Carlsbad, CA) and an miRNeasy Serum/Plasma Kit (Qiagen, Hilden, Germany) according to the manufacturers’ instructions.

**Cell culture and *in vitro* experiments**

Human CRC cell lines (HCT15, SW620, SW480 and LOVO) and 293T were purchased from American Type Culture Collection (ATCC) (Manassas, VA). All of the cells were authenticated, and no mycoplasma contamination was detected (data not shown). Cell lines were maintained in DMEM supplemented with 10% FBS, 100 units penicillin and streptomycin per ml, and cultured at 37℃ in 5% CO2. For hypoxia treatments, we prepared extracts in a hypoxia chamber (I-Glove, BioSpherix, Parish, NY).

**Lentivirus stable transduction**

Lentiviruses were generated by co-transfection of the expression vector of interest with the packaging plasmids psPAX2 and pMD2G. The day before transduction, the HCT15, SW620, SW480, LOVO cells were trypsinized, 2×10^5^ cells per well plated in 1 mL complete culture medium in a six-well plate, and incubated overnight at 37°C. On the day of transduction, the medium was removed and replaced with 1 mL of complete medium with 10 µg/mL polybrene (Cat.No.107689, Sigma, St. Louis, MO). Adenovirus particles were thawed at room temperature, mixed gently, and added to the HCT15, SW620, SW480, and LOVO cells. After gently mixing, cells were incubated overnight. After 12 hours, culture medium was replaced with 2 mL of complete medium containing 10 µg/mL blasticidin S (Cat. No. ant-bl, Invitrogen, San Diego, CA), which was replaced every 2 days for 1 week after all of the control cells had died. Positive cells were propagated in 1 µg/mL blasticidin S for 2 weeks, and then frozen until use. For experiments, cells were thawed and allowed to grow for three passages before use.

**Western blotting**

Total proteins were isolated from tissues using a total protein extraction kit (Keygen, Nanjing, China). A total of 40 µg of protein was separated using sodium dodecylsulfate-polyacrilamide gel electrophoresis (SDS-PAGE) and transferred onto polyvinylidene difluoride (PVDF) membranes and then blocked with 5% fat-free milk at room temperature for 2 hours. The immune-blot was incubated with primary antibody detecting vimentin and E-cadherin (1: 1000 dilution; cell signaling), and β-actin (1:1000 dilution; cell signaling) was used as a control. The signals were detected using a Super ECL Plus Kit (Keygen) and determined by quantitative analysis using UVP software (UVP, LLC, Upland, CA, United States).

**siRNA, shRNA, and Plasmid Construction and Cell Transfection**

siRNAs targeting circPTK2 and vimentin were designed and synthesized by RiboBio (Guangzhou, China).For siRNA knockdown, we transfected CRC cells in a six-well plate with siRNAs. Transfection was performed with Lipofectamine 2000 (Invitrogen, USA) or Lipofectamine RNAiMAX (Invitrogen) according to the manufacturer’s protocols. shRNAs targeting circPTK2 were designed based on the siRNA sequences and were cloned into the pGLVH1/GFP+Puro vector (GenePharma, Shanghai, China); the two shRNAs that produced the best knockdown efficiencies were used in follow-up functional research. The circPTK2-overexpressing lentivirus plasmid was synthesized by GenePharma (Shanghai, China).

**Analysis of cell colony formation, invasion, migration ability, and apoptosis**

In brief, for colony formation assays, 500 cells were seeded in 10-cm plates and allowed to grow in complete medium for 10 days. For the cell invasion assays, 1×10^5^ cells were plated into the Transwell insert coated with Matrigel (CytoSelect 24-Well Cell Invasion Assay Kit; Cell Biolabs, USA) and cultured with complete medium for 48 hours. For cell migration assays, 1×10^5^ cells were plated into the Transwell insert and cultured with complete medium for 24 hours. Finally, all cells were fixed with ethanol and stained with crystal violet. For detection of apoptosis, cells were stained with annexin V-FITC/PI at room temperature in the dark. The rate of apoptosis was analyzed by flow cytometry. All assays were independently performed in triplicate.
